# Determinants of generic drug substitution in Switzerland

**DOI:** 10.1186/1472-6963-11-17

**Published:** 2011-01-26

**Authors:** Anne Decollogny, Yves Eggli, Patricia Halfon, Thomas M Lufkin

**Affiliations:** 1 Institute of Health Economics and Management, Centre Hospitalier Universitaire Vaudois and University of Lausanne, Route de Chavannes 31, 1015 Lausanne, Switzerland; 2 Institute of Social and Preventive Medicine, Centre Hospitalier Universitaire Vaudois and University of Lausanne, Rue du Bugnon 17, 1005 Lausanne, Switzerland; 3 Institute of Health Economics and Management, Faculty of Business and Economics, University of Lausanne, Route de Chavannes 31, 1015 Lausanne, Switzerland

## Abstract

**Background:**

Since generic drugs have the same therapeutic effect as the original formulation but at generally lower costs, their use should be more heavily promoted. However, a considerable number of barriers to their wider use have been observed in many countries. The present study examines the influence of patients, physicians and certain characteristics of the generics' market on generic substitution in Switzerland.

**Methods:**

We used reimbursement claims' data submitted to a large health insurer by insured individuals living in one of Switzerland's three linguistic regions during 2003. All dispensed drugs studied here were substitutable. The outcome (use of a generic or not) was modelled by logistic regression, adjusted for patients' characteristics (gender, age, treatment complexity, substitution groups) and with several variables describing reimbursement incentives (deductible, co-payments) and the generics' market (prices, packaging, co-branded original, number of available generics, etc.).

**Results:**

The overall generics' substitution rate for 173,212 dispensed prescriptions was 31%, though this varied considerably across cantons. Poor health status (older patients, complex treatments) was associated with lower generic use. Higher rates were associated with higher out-of-pocket costs, greater price differences between the original and the generic, and with the number of generics on the market, while reformulation and repackaging were associated with lower rates. The substitution rate was 13% lower among hospital physicians. The adoption of the prescribing practices of the canton with the highest substitution rate would increase substitution in other cantons to as much as 26%.

**Conclusions:**

Patient health status explained a part of the reluctance to substitute an original formulation by a generic. Economic incentives were efficient, but with a moderate global effect. The huge interregional differences indicated that prescribing behaviours and beliefs are probably the main determinant of generic substitution.

## Background

Generic substitution has been associated with notable monetary savings for society in several settings [1] and represents one of several strategies aimed at curb pharmaceutical expenditure [2-4]. Generic drugs, which contain the same therapeutic substance as the original formulation, become available once the patent protection granted to the brand name drug has expired, leading to greater market competition and lower prices [5]. To contain rising pharmaceutical costs, governments and health insurers should do more to promote generic substitution.

There are, however, different barriers to the wider use of generic drugs. The first is the concern of patients. About one third of patients expressed worries after generic substitution and some reported either a reduced effect or new or increased side-effects [6,7]. Chronically ill patients taking several drugs may feel unsettled [8], particularly when different generics are offered each time they buy their medication [9,10]. Such brand-to-generic or generic-to-generic switches might be confusing (patients taking the same substance but in a new form), and problematic for certain medication classes with a narrow therapeutic margin like anti-epileptics, where seizures and other negative outcomes have been reported [11,12]. Generic substitution could be an additional factor behind poor therapy adherence in chronic diseases [10].

Furthermore, generic substitution is generally met with skepticism by health professionals despite a lack of proven differences in the clinical outcomes of generics and original formulations [13,14]. Physicians who play a central role in the prescription decision have their individual prescribing habits [15] and tend to prescribe by brand name, generally ignoring drug prices [16,17]. Pharmacies may also influence the choice of medication by informing patients of the costs or by adopting procedures that increase generic use.

Finally, economic and regulatory conditions play a major role on the drugs market, with financial incentives for all parties (prescribers, pharmacists, and patients) being an important factor [2]. Patients who face higher co-payments purchase more generics on average, and they switch to a generic when the relative saving is high [18]. Market characteristics, as well as pricing and licensing policies also influence the use of generic drugs. The market share of generics varies widely from one country to another [19]. In markets where the generics' share is large, switching should be more commonplace. However, brand-name drugs tend to be heavily advertised and prescribers tend to remain loyal to brands, allowing them to keep their customers for long periods despite being more expensive [14].

The aim of this study was to explore the relationship between the use of generic drugs and its main determinants, i.e. patients, physicians, and certain characteristics of the generics' market to adapt if necessary policies on this area.

We used reimbursement claims data from a Swiss health insurer for the year 2003. This period was chosen specifically because it came two years after the introduction of new generic substitution rules in Switzerland and preceded a reform imposing a minimum price difference between original formulations and generics. In addition, drug prices were relatively stable during that year. In the interests of identifying factors that could lead to more efficient prescribing, three cantons were selected, one from each linguistic region of Switzerland.

## Methods

### Setting

Until 2005 Switzerland had the second smallest market share of generic drugs in Europe and, until recently, the generic substitution rate was well below the average [20]. Two thirds of drugs were reimbursed by the compulsory health insurance system, and accounted for more than 20% of health insurers' expenditures.

It is compulsory for all Swiss residents to purchase a health insurance contract. All health insurance funds offer the same package, as set down by the federal authorities, which features a list of reimbursable prescription drugs. Swiss patients contribute to the cost of care through the payment of an annual deductible. They are free to choose the level of their deductible, but the higher the deductible the lower the premium they pay. In 2003, there was no mandatory deductible for children under 18, and a minimum deductible of 230 Swiss francs for adults. The highest was 375 Swiss francs for children and 1,500 Swiss francs for adults. Furthermore, once this is reached, the insured is subject to a co-payment of 10% of the cost of any care service he has received. Co-payments are capped at an annual ceiling depending on the deductible (300 to 2,100 Swiss francs). This means that insurance contracts only differ in terms of the financial incentives associated with the deductible levels, and not in the types of care or drugs that are covered. All patients therefore face the same choice of branded or generic drugs for a given indication.

### Recent generic medicine policy implemented in Switzerland

Since January 2001, pharmacists have been authorized to substitute original drugs with generics provided that they have the agreement of the patient and have informed the prescribing physician. Since July of the same year pharmacists have received a fixed fee when dispensing a generic drug, independent of the price of the dispensed drug, which avoids financial penalization. There is no reference pricing scheme that requires the patient to pay the difference between the actual price of the medicine and the reference price. However, since 2006, patient copayments for brand drugs have been raised from 10% to 20%. To be admitted to the reimbursed drugs list, generics need to be priced lower than the branded drug. This means a minimal saving in comparison with the originator being requested since 2005 (in 2009, -20% to -50%, depending on market size). Switzerland has no other mechanisms in place like mandatory guidelines and expenditure targets to regulate physicians' generic prescription practices.

### Studied population

The data used in this study were on the drugs' reimbursement claims submitted by the entire population of insured of one of Switzerland's largest health insurers (CSS), who lived in one of three cantons - Aargau, Ticino and Vaud - during 2003. In Switzerland, most drugs are delivered through medical prescriptions, but some over-the-counter (OTC) drugs are also reimbursed if prescribed by a physician.

This source population of 169,837 subjects represented 15.6%, 10.4%, 7.6% of the population of these cantons respectively. Each canton reflects one of Switzerland's three main linguistic regions (German-speaking Aargau, French-speaking Vaud and Italian-speaking Ticino), and none allows physicians to dispense drugs [21]. Those insured with CSS were slightly older than the general Swiss population (14% of insured were 70 years old or more, against 11% in the general population during the same year) but their gender distribution was similar. Nevertheless, the odds ratios were adjusted for age and gender, allowing a generalization of the observed associations to broader populations. Indeed, it was highly improbable that there was any association between the studied effects and characteristics of the sample populations. In particular, there is no evidence that those insured with the CSS had different incentives than others, because in 2003 HMOs and managed care services were scarce in Switzerland and also in the three cantons.

The studied population consisted of all observations relating to substitutable drugs. To be considered substitutable, a drug must have had at least one generic and one brand-name counterpart sold in Switzerland and was reimbursable by the compulsory basic health insurance system in 2003. Counterpart here means the same active substance, pharmaceutical form (tablets, drops, etc.), dosage, administration route (oral, inhalation, etc.), similar packaging size (differences in number of doses lower than 30%) and intended use as defined in the Swiss Medicines Compendium [22]. Each substitutable drug was thus allocated to a substitution group which was identified by the branded original and comprised all its corresponding generic products. Our dataset comprised a total of 298 substitution groups, generics corresponding to 20.4% of the total quantity of drugs delivered in 2003. The observation unit was one drug prescribed to an insured patient by a physician and dispensed by a pharmacist. A renewable prescription (same drug prescribed by the same physician) was counted only once if it was dispensed by the same pharmacist, as it was considered to be the consequence of the same decision. To avoid the inclusion of exceptional prescribing situations, we only considered drugs that were prescribed by a physician and dispensed by a pharmacist to a patient resident in the same canton.

### Outcome

The study outcome was to ascertain whether the patient had received a generic drug or not.

### Predictors of generic drug use

**Figure 1:**
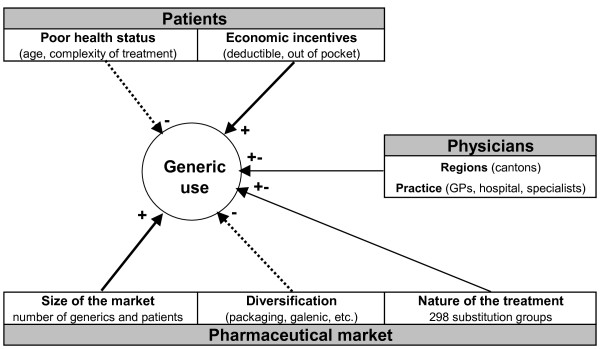
**Conceptual framework and assumed determinants of generic use**.

Theoretically, there are at least three main promoting (+) or hindering (-) determinants of the use of generics: patients, physicians and the drugs' market, (Figure 1).

As mentioned in the background section, patients with poor health status (the old, complex treatments) tend to be reluctant to use generic drugs. However, in a co-payment setting (high deductible and out-of-pocket participation), patients tend to prefer saving money, and are likely to choose a generic over a branded drug, especially if the price of the latter is much higher than that of the former. Patients were characterized by demographic variables (gender and age categories: 0-19, 20-39, 40-59, 60-79, and 80+), by treatment complexity which was represented by the number (1-5, 6-10, 11-15, 16-20, 21+) of the 4^th ^level (chemical subgroups) of Anatomical Therapeutic Chemical (ATC) codes of dispensed drugs (chemical subgroup level), by whether they had chosen an optional health insurance deductible over the standard minimum deductible, by out-of-pocket participation (more precisely by whether the financial participation of the patient remained under the maximum participation amount, comprising the deductible and co-payments), and by the canton of residence (Aargau, Ticino or Vaud). Treatment complexity can be measured by the ATC drug classification system because dispensed drugs are a direct measure of treated morbidity and have already been used as a risk adjustment tool, particularly when diagnostic information is not available [23]. A simple count of dispensed drugs in the current year would overestimate the disease burden. Several similar active ingredients (i.e. two anti-hypertensives in the same therapeutic class) reflect rather different effects (adverse events or efficacy) other than the disease severity. Several drugs in different therapeutic chemical subgroups, such as antihypertensives with different mechanisms of action, reflect a more serious condition.

Given that physicians write prescriptions, they heavily influence the choice of medication. Since our study focused on three cantons which did not have significant drug dispensation by physicians, the latter had no financial interest in the choice of drugs and were assumed to be "loyal agents" acting in the interests of their patients. It was therefore difficult to formulate a priori hypotheses about their influence. Prescribing physicians' variables were their practice status (hired by a hospital, independent specialist, independent general physician or internist), and the canton where they worked.

The only information on pharmacies was the canton where they were located. This lack of information should not compromise the study, because Swiss pharmacists had no significant financial disincentives to dispense generics. Since we excluded out-of-canton drug dispensation, the canton variable was the same for patients, physicians and pharmacies, and was thus included as a separate category and only once.

There is no evidence to support the assumption that the size of the generic market has a bearing on generics' substitution. On the contrary, launching new galenic and packaging forms were found to be effective mechanisms for maintaining consumption of brand name drugs.

The substitution groups were characterized by the number of different generic drugs available in Switzerland in 2003 (1-2, 3-4, 5-6, and 7+), the number of patients treated (1-1000, 1001-2000, 2001-3000, and 3,000+), the unit price of the original (expressed in quintiles), the relative price difference (the price of the original per unit minus the price of the cheapest drug in the group divided by the price of the original, expressed in quintiles), the administration mode (oral, topical, nasal, inhalation or ophthalmic preparations; others), whether there were multiple packaging sizes, whether there was an additional galenic form for a branded drug (for example effervescent, sublingual or extended release medication), whether the group had at least one co-branded original (product sold under license or a copy of the original with different packaging, such as blister pack, box or bottle). Repackaging and reformulating a brand-name drug is a common technique used to maintain the attractiveness of the originals product. To avoid a collinear association with the price of the medication, we used the number of patients treated in each substitution group rather than the total amount of sales as an indicator of the size of the market.

Finally, we introduced the 298 substitution groups as dummy variables to identify the possible influence of other aspects of the treatments.

### Statistical model

The aim of the analysis was to separate the factors related to the dispensing procedure (such as patient or physician characteristics) from the characteristics of the substitution groups depicting the drugs market. We used three logistic models. The simpler model (Model A) comprised only the patient and physician variables and the cantons. However, we did include some first-order interactions between the variables.

We then added dummy indicators of the substitution groups, which take the value 1 if the observation (the drug) belongs to the substitution group and 0 if not (Model B). Finally, the third model (Model C) includes all the variables of Model A and all the variables describing the substitution groups.

The area under the receiver operating characteristic (ROC) curve (C-statistics) was used to assess the discriminatory power of the predictive model and the Hosmer-Lemeshow table to test its calibration. Akaike's information criterion (AIC) and Schwarz's Bayesian information criterion (BIC) were used to compare the competing models [24].

To estimate how different policies could influence generic drug use, we computed the marginal effects of all the variables from Model C (the most complete model). They were computed as discrete differences from the reference category (zero for binary variables, and as mentioned in the table for categorical variables). For example, the marginal effect of the variable is the difference between the probability of generic drug use when everyone is considered to be between 0 and 19 years old, and the probability when everyone is considered as to be between 60 and 79 years old, while all other variables hold constant. The computation was not possible for the administration mode and the three character ATC code due to missing cells, caused by the fact that not all administration modes are compatible with all ATC codes.

All statistical analyses were performed using Stata 11.0 (Stata Corp LP, College Station, Texas, USA).

## Results

The source population included 1,341,197 drug dispensations, among which 273,797 were eligible for generic prescription. This population fell to 186,569 after aggregating multiple deliveries and to 173,212 once out-of-canton prescriptions (physicians, pharmacist or patients from a different canton) were excluded.

### Univariate analysis

Descriptive statistics and the results of the univariate analysis (logit model with only one variable and a constant as explanatory variables) are given in Table 1. The overall substitution rate of generics was 31%, with considerable differences across the three cantons (Aargau: 43%, Ticino: 12%, Vaud: 35%). Low substitution rates (odds ratios significantly < 1) were associated with female and older patients, optional health insurance deductibles, treatment complexity (more than 10 different drugs delivered in the same year), hospital prescribers, cheaper brand medication, non-oral administration modes, a substitution group consisting of an original with a special galenic form or involving at least one product sold under license. Higher rates were observed among young patients, those subject to out-of-pocket participation, substitution groups containing many generics or many patients, large relative differences in price or varying packaging sizes.

**Table 1:** Descriptive statistics and univariate logit estimates

	Frequency	Substitution rate	Odds ratio	95%CI
OUTCOME VARIABLE Use of a generic drug (yes = 1, no = 0)	0.313			
CANTONS				
Aargau (reference)	0.372	0.433		
Vaud	0.329	0.350	0.706	[0.689, 0.722]
Ticino	0.299	0.124	0.186	[0.181, 0.192]
PATIENTS' VARIABLES Female:				
no (reference)	0.378	0.317		
yes	0.622	0.311	0.972	[0.952, 0.993]
Age:				
0-19	0.100	0.411	2.057	[1.986, 2.131]
20-39	0.158	0.410	2.050	[1.989, 2.112]
40-59	0.250	0.344	1.543	[1.502, 1.585]
60-79 (reference)	0.356	0.254		
80+	0.136	0.228	0.868	[0.838, 0.900]
Optional health insurance deductible:				
no (reference)	0.619	0.344		
yes	0.381	0.264	0.685	[0.671,. 0.700]
Out-of-pocket participation:				
no (reference)	0.396	0.241		
yes	0.604	0.361	1.777	[1.740, 1.816]
Treatment complexity:				
1-5 drugs	0.215	0.424	1.429	[1.390, 1.469]
6-10 drugs (reference)	0.290	0.340		
11-15 drugs	0.223	0.280	0.757	[0.735, 0.779]
16-20 drugs	0.139	0.246	0.634	[0.613, 0.657]
21+ drugs	0.133	0.202	0.491	[0.473, 0.509]
PHYSICIANS' VARIABLES Specialist prescriber:				
no (reference)	0.777	0.314		
yes	0.223	0.310	0.978	[0.955, 1.003]
Hospital prescriber:				
no (reference)	0.880	0.325		
yes	0.120	0.225	0.603	[0.583,.0.624]
SUBSTITUTION GROUPS' VARIABLES Number of generics				
1-2 (reference)	0.333	0.153		
3-4	0.260	0.374	3.322	[3.225, 3.422]
5-6	0.298	0.415	3.934	[3.823, 4.049]
7+	0.110	0.382	3.430	[3.305, 3.559]
Number of treated patients:				
1-1000 (reference)	0.361	0.263		
1001-2000	0.259	0.322	1.334	[1.299, 1.370]
2001-3000	0.122	0.223	0.803	[0.774, 0.834]
3001+	0.257	0.418	2.017	[1.965, 2.070]
Price of brand medication:				
1st quintile	0.189	0.327	1.000	[0.969, 1.031]
2nd quintile	0.165	0.251	0.689	[0.666, 0.713]
3rd quintile (reference)	0.239	0.327		
4th quintile	0.187	0.328	1.002	[0.972, 1.034]
5th quintile	0.220	0.321	0.972	[0.943,.1.001]
Relative price difference:				
1st quintile	0.188	0.200	0.599	[0.578, 0.620]
2nd quintile	0.200	0.347	1.267	[1.227, 1.308]
3rd quintile (reference)	0.206	0.295		
4th quintile	0.204	0.301	1.029	[0.996, 1.062]
5th quintile	0.203	0.416	1.701	[1.649, 1.754]
Administration mode:				
Oral (reference)	0.839	0.328		
Topic	0.095	0.285	0.817	[0.788, 0.847]
Inhalation, nasal & ophthalmic	0.032	0.046	0.099	[0.087, 0.112]
Parenteral, rectal & vaginal	0.034	0.273	0.769	[0.726, 0.815]
Original with special galenic form:				
no (reference)	0.862	0.338		
yes	0.138	0.160	0.373	[0.359, 0.386]
At least one drug sold under license:				
no (reference)	0.818	0.316		
yes	0.182	0.300	0.929	[0.904, 0.954]
Multiple package sizes:				
no (reference)	0.575	0.294		
yes	0.425	0.339	1.232	[1.207, 1.258]

Observations	173,212			

### Multivariate analysis

The results of the multivariate analysis are given in Table 2.

**Table 2:** Determinants of generics use (logistic regression)

	Model A Cantons and patients variables	Model B Model A + substitution groups dummies	Model C Model A + substitution groups variables
	Coeff. [95%CI]	Coeff. [95%CI]	Coeff. [95%CI]
CANTON:			
Aargau (reference)			
Vaud	-0.253 [-0.31,-0.20]	-0.247 [-0.31,-0.19]	-0.248 [-0.31,-0.19]
Ticino	-1.391 [-1.46,-1.32]	-1.459 [-1.53,-1.39]	-1.463 [-1.53,-1.39]
PATIENTS' VARIABLES Female	0.023 [0.00,0.05]	0.031 [0.01,0.06]	0.017 [-0.01,0.04]
Age:			
0-19	0.340 [0.24,0.44]	0.238 [0.12,0.36]	0.207 [0.09,0.32]
20-39	0.291 [0.22,0.36]	0.101 [0.02,0.18]	0.127 [0.05,0.21]
40-59	0.237 [0.18,0.30]	0.158 [0.09,0.22]	0.164 [0.10,0.23]
60-79 (reference)			
80+	-0.135 [-0.21,-0.06]	-0.018 [-0.10,0.06]	-0.041 [-0.12,0.04]
Age 0-19 in Vaud	0.199 [0.12,0.28]	-0.004 [-0.10,0.09]	0.086 [-0.00,0.18]
Age 20-39 in Vaud	0.026 [-0.05,0.10]	0.055 [-0.02,0.14]	0.051 [-0.03,0.13]
Age 40-59 in Vaud	-0.058 [-0.12,0.01]	-0.051 [-0.12,0.02]	-0.046 [-0.11,0.02]
Age 80+ in Vaud	0.185 [0.10,0.27]	0.194 [0.10,0.29]	0.211 [0.12,0.30]
Age 0-19 in Ticino	-0.437 [-0.58,-0.30]	-0.548 [-0.70,-0.40]	-0.484 [-0.63,-0.34]
Age 20-39 in Ticino	-0.160 [-0.26,-0.06]	-0.176 [-0.28,-0.07]	-0.168 [-0.27,-0.07]
Age 40-59 in Ticino	-0.208 [-0.29,-0.13]	-0.192 [-0.28,-0.11]	-0.189 [-0.27,-0.10]
Age 80+ in Ticino	0.095 [-0.01,0.20]	0.075 [-0.03,0.18]	0.098 [-0.01,0.20]
Optional health insurance deductible	0.065 [0.04,0.09]	0.039 [0.01,0.07]	0.043 [0.02,0.07]
Out-of-pocket participation	0.233 [0.18,0.28]	0.146 [0.09,0.20]	0.178 [0.12,0.23]
Out-of-pocket participation at age 0-19	0.084 [-0.02,0.19]	0.069 [-0.04,0.18]	0.043 [-0.07,0.15]
Out-of-pocket participation at age 20-39	0.205 [0.13,0.28]	0.165 [0.08,0.25]	0.139 [0.06,0.22]
Out-of-pocket participation at age 40-59	0.052 [-0.01,0.11]	-0.020 [-0.09,0.05]	-0.025 [-0.09,0.04]
Out-of-pocket participation at age 80+	-0.161 [-0.24,-0.09]	-0.150 [-0.23,-0.07]	-0.136 [-0.22,-0.05]
Out-of-pocket participation in Vaud	-0.062 [-0.12,-0.01]	-0.066 [-0.12,-0.01]	-0.067 [-0.12,-0.01]
Out-of-pocket participation in Ticino	-0.107 [-0.18,-0.04]	-0.097 [-0.17,-0.02]	-0.116 [-0.19,-0.04]
Treatment complexity:			
1-5 drugs	0.112 [0.08,0.14]	0.096 [0.06,0.13]	0.082 [0.05,0.11]
6-10 drugs (reference)			
11-15 drugs	-0.063 [-0.09,-0.03]	-0.055 [-0.09,-0.02]	-0.055 [-0.09,-0.02]
16-20 drugs	-0.089 [-0.13,-0.05]	-0.101 [-0.14,-0.06]	-0.102 [-0.14,-0.06]
21+ drugs	-0.186 [-0.23,-0.14]	-0.175 [-0.22,-0.13]	-0.174 [-0.22,-0.13]
PHYSICIANS' VARIABLES Specialist prescriber	-0.245 [-0.27,-0.22]	-0.203 [-0.23,-0.17]	-0.194 [-0.22,-0.16]
Hospital prescriber in Aargau	-0.871 [-0.93,-0.81]	-1.125 [-1.19,-1.06]	-1.101 [-1.17,-1.04]
Hospital prescriber in Vaud	-0.344 [-0.40,-0.29]	-0.559 [-0.62,-0.50]	-0.571 [-0.63,-0.51]
Hospital prescriber in Ticino	-0.737 [-0.84,-0.63]	-0.778 [-0.89,-0.67]	-0.756 [-0.86,-0.65]
SUBSTITUTION GROUPS' VARIABLES Number of generics:			
1-2 (reference)			
3-4			0.506 [0.46,0.55]
5-6			0.745 [0.70,0.79]
7+			0.522 [0.46,0.58]
Number of treated patients:			
1-1000 (reference)			
1001-2000			0.109 [0.07,0.15]
2001-3000			0.412 [0.35,0.47]
3001+			0.013 [-0.03,0.06]
Price of brand medication:			
1st quintile			0.181 [0.13,0.23]
2nd quintile			-0.006 [-0.07,0.05]
3rd quintile (reference)			
4th quintile			0.072 [0.03,0.12]
5th quintile			-0.064 [-0.12,-0.01]
Relative price difference:			
1st quintile			-0.303 [-0.35,-0.25]
2nd quintile			-0.167 [-0.21,-0.12]
3rd quintile (reference)			
4th quintile			0.026 [-0.02,0.07]
5th quintile			0.219 [0.16,0.28]
Administration mode: Oral (reference)			
Topic			-0.644 [-0.71,-0.57]
Inhalation, nasal & ophthalmic			-1.329 [-2.05,-0.61]
Parenteral, rectal & vaginal			-0.676 [-0.76,-0.59]
Original with special galenic form			-0.476 [-0.55,-0.41]
At least one drug sold under license			-0.309 [-0.36,-0.26]
Multiple package sizes			-0.233 [-0.27,-0.19]
Constant	-0.554 [-0.60,-0.50]	0.717 [0.64,0.80]	-0.289 [-0.38,-0.20]
Substitution groups dummies	No	Yes	No
3 char. ATC codes dummies	No	No	Yes

Pseudo-R-squared	0.089	0.229	0.194
AIC	196,349	166,597	173,677
BIC	196,671	169,867	174,542
Area under ROC curve	0.699	0.811	0.791
Hosmer-Lemeshow chi2	4.295	101.676	139.595
Prob > chi2	0.830	0.000	0.000
Observations	173,212	173,212	173,212

A multivariate logistic regression with only the patients' variables and cantons gave results similar to the univariate analysis, except for the "deductible" variable (Table 2, Model A). Higher deductibles were associated with lower generic substitution in the univariate analysis but higher rates in the multivariate analysis. One explanation for this finding was confounding by age. The youngest had the highest substitution rates regardless of their deductible and more frequently had a standard deductible (>90%) than adults (<60%). Other factors such as treatment complexity might influence both the choice of deductible and the substitution behavior. Among the oldest age group, out-of-pocket participation favored generic use much less. Cantons exerted the strongest influence on substitution rates. The influence of age on substitution rates also differed across cantons. In the canton of Vaud, substitution rates tended to be higher for the youngest and lower for the oldest. The inverse correlation between age and substitution rates was less pronounced in Ticino, where rates were uniformly low. Generic use among hospital physicians was particularly low in Ticino. In both Ticino and Vaud, out-of-pocket participation had a lower incentive effect on substitution.

Model A passed the Hosmer and Lemeshow [25] goodness of fit test, with a p-value of 0.83.

Taking the heterogeneity of the substitution groups into account did not change the observed trends significantly, but improved the discriminatory power of the model. The C-statistics moved from less than 70% to over 81% (Table 2, Model B), thus providing evidence that the characteristics of a given generic drug have a significant bearing on the substitution rate.

The replacement of substitution groups by all their measurable characteristics provide a model of comparable predictive performance with a 79% C-statistics (Table 2, model C). Substitution rates for non-oral formulations were always significantly lower than pills (all coefficients significantly < 0). Market-related variables had a considerable effect on substitution rates. The number of patients treated, the number of generics offered on the market, and the relative savings with the substitutable product increased substitution rates. Duplicates of original drugs (galenic form and drugs sold under license and multiple package sizes) acted as barriers to substitution.

The Hosmer and Lemeshow tests for Models B and C indicated a lack of fit. This was due mainly to missing interaction effects between the cantons and the group identifiers in Model B and the market variables in Model C. We excluded these effects in order to keep the models tractable. Moreover, for very large samples like ours, the Hosmer-Lemeshow test is often oversensitive to detect lack of fit [26].

The marginal effects of each factor are reported in Table 3. The negative impact of health status was rather weak: the combined effect of old age and treatment complexity did not exceed a 3% difference in substitution rates. Although the deductible amount and cost participation were associated with an increased use of generics, their effects were moderate: less than 1% for a non-standard deductible and 2% for patients who did not reach their maximum out-of-pocket participation. In contrast, generic market share strongly influenced generic use. Introducing new generics on the market would increase the substitution rate from 8 to 12%. One thousand patients more increased use from about 2% to 7% till 3,000 patients. Each additional quintile of relative price savings contributes on average an additional 1.5% substitution. However, the diversification of brand drug supply through minor modifications had a strong negative effect on generic use (galenic form -7%, additional patent -5%, multiple packaging size -4%).

**Table 3:** Average marginal effects (logistic regression - Model C)

	Marginal effect	95%CI
CANTON		
Aargau (reference)		
Vaud	-0.040	[-0.045 -0.034]
Ticino	-0.260	[-0.265 -0.255]
PATIENTS' VARIABLES Female	0.003	[-0.001 0.007]
Age:		
0-19	0.031	[0.022 0.040]
20-39	0.035	[0.028 0.041]
40-59	0.016	[0.011 0.022]
60-79 (reference)		
80+	-0.005	[-0.013 0.002]
Optional health insurance deductible	0.007	[0.003 0.012]
Out-of-pocket participation	0.023	[0.018 0.028]
Treatment complexity:		
1-5 drugs	0.014	[0.009 0.020]
6-10 drugs (reference)		
11-15 drugs	-0.009	[-0.015 -0.004]
16-20 drugs	-0.017	[-0.024 -0.010]
21+ drugs	-0.029	[-0.037 -0.021]
PHYSICIANS' VARIABLES Specialist prescriber	-0.032	[-0.037 -0.027]
Hospital prescriber	-0.131	[-0.137 -0.125]
SUBSTITUTION GROUPS' VARIABLES Number of generics:		
1-2 (reference)		
3-4	0.083	[0.075 0.090]
5-6	0.125	[0.118 0.133]
7+	0.085	[0.076 0.095]
Number of treated patients:		
1-1000 (reference)		
1001-2000	0.018	[0.012 0.025]
2001-3000	0.071	[0.060 0.082]
3001+	0.002	[-0.006 0.010]
Price of brand medication:		
1st quintile	0.031	[0.023 0.039]
2nd quintile	-0.001	[-0.011 0.009]
3rd quintile (reference)		
4th quintile	0.012	[0.004 0.020]
5th quintile	-0.011	[-0.019 -0.002]
Relative price difference:		
1st quintile	-0.050	[-0.058 -0.042]
2nd quintile	-0.028	[-0.036 -0.020]
3rd quintile (reference)		
4th quintile	0.004	[-0.004 0.013]
5th quintile	0.038	[0.028 0.048]
Original with special galenic form	-0.077	[-0.088 -0.066]
At least one drug sold under license	-0.051	[-0.058 -0.043]
Multiple package sizes	-0.039	[-0.045 -0.032]

Notes:

* p < 0.05 ** p < 0.01 *** p < 0.001

The marginal effect for discrete and factor variables is the discrete change from the reference level

Prescribing practices, which were measured across hospital physicians, specialist physicians and cantons, had the strongest impact on generic substitution. The substitution rate in the canton of Ticino was much lower (-26%) than in Aargau. The rate difference between hospital physicians and specialists was -13% and -3% between hospital and general physicians.

Among substitutable drugs, only one was included in the list of narrow therapeutic index published by the US Food and Drug Administration [27]: carbamezepine, which was at the 13^th ^percentile of substitution odds (rather low).

## Discussion

According to our data, generics' prescriptions in 2003 accounted for only 20% of the overall prescription volume. In contrast, generics accounted for more than 40-60% in some OECD countries [5], even though the generics markets are not totally comparable. It is therefore possible that our results could not be replicated exactly in other countries due to differences in the nature of the generics' market.

We found that the variations in substitution rates across the cantons were similar to those observed across European countries (36% in Germany, 8% in France and 4% in Spain, for instance) during the same period. Adjusting for potential disparities in health status, prescriptions or prescribers' characteristics did not alter these variations, which suggests that the impact of cultural aspects such as prescribing behaviours or beliefs about generic substitution may be as important as national policies. This interpretation is confirmed by the finding that hospital physicians substituted less, and should therefore be a focus of any future initiatives encouraging more effective outpatient prescribing practices.

As shown in previous studies, poorer health status, captured in the present study by age and treatment complexity, was associated with lower generic use. Older people are less willing to substitute when offered the choice [28]. This result is congruent with another study on hypertensive patients, which found that substitution had generated poor adherence, as well as worries and confusion regarding the new treatment, especially when more than one equivalent generic drug was used [10]. For instance, a clear and conspicuous indication of the prescribed substance that is the same for both the branded and the generic drug could improve patient confidence.

Pricing and reimbursement regulations thus appear to be of utmost importance when it comes to increasing generic use. Consumers facing high out-of-pocket costs bought more generics. They were more likely to substitute generics when the savings relative to brand name price would be high. However, the marginal effects of financial incentives related to insurance coverage were rather weak in our study. This could be explained by the annual ceiling of patients' co-payments that protect people against high out-of-pocket expenses. Another reason could be the relatively low proportion of pharmaceutical costs in Switzerland (less than 10% of total out-of pocket health expenditures against 19 to 65% in 12 OECD countries [20] when OTC products are included). In 2006, the co-insurance rate increased to 20% instead 10% for brand-name drugs for which less expensive interchangeable generics are available. Minimum costs savings rate per product were also introduced to boost generic use. It would be interesting to assess the impact of these two changes on the propensity towards generic use.

The analysis of the substitution market generated interesting additional observations.

First, we found that more generics' competitors, a large market and more potential cost savings for consumers increased generic use. In Switzerland, administrative constraints (such as the translation of labels in the three official languages), high patent protection and the small market size may thus explain the low generic market share.

Second, measures to protect brand name exclusivity such as reformulations and repackaging were also effective in curbing generic distribution. Although these minor changes may confer benefits on some patients, it is also possible that others may be switched inappropriately to these reformulated drugs, whose prices are higher than the generics. For instance, a recent meta-analysis on cardiovascular drugs found no evidence to support the claim that these peripheral differences between brand name and generic drugs (such as inert binders, fillers, or specific manufacturing processes) constitute a significant clinical advantage [29].

Our study has limitations. The source population was limited to a single, large insurance company and it is therefore possible that the population was not totally representative of the Swiss population. Another issue is related to the non-reimbursement of dispensed drugs for patients who have not asked for reimbursement. The actual substitution rate on the Swiss generics market therefore could be slightly higher than observed. Furthermore, our results could not reflect the behaviour of patients in relatively good health who did not reach the deductible. After adjustment of measurable factors, there remained large differences in mean substitution rates among patients, physicians and pharmacies. Further qualitative research would be useful to identify modifiable behavioral determinants.

Nevertheless, our findings highlight that policies on pricing and reimbursement are important as an impetus for the generic medicines market but not sufficient for a sustained generic market share. The opposite effects of stimulating the substitution market or protecting brand products provide some evidence that generic substitution is more than a simple question of time.

The policy measures recently introduced in Switzerland - 20% co-payment for brand drugs, minimal savings between originals and generics - should be effective since the relative price difference and out-of-pocket participation variables were both significantly associated with a higher rate of generic use. However, if all existing incentives under the control of health policies were fully applied, i.e. maximizing patients' contribution to the costs of care through deductibles and out-of-pocket payments and increasing the number of generics on the market, they would provide an additional potential switch towards generics of about 10% (see Table 3: patient contribution +3.0%, switching from 1-2 generics to 3-4 + 8.3%). Additional pricing and reimbursement rules should be introduced in Switzerland to boost generic drug substitution to the levels observed in other OECD countries. In particular, the introduction of a nationwide reference pricing scheme should be discussed. Wide differences betweens counties suggest that educational approaches targeted at physicians should be investigated with a view to increasing their confidence in generics' efficacy and safety.

The relatively low substitution rate observed for carbamazepine could be explained by the reluctance of physicians to prescribe drugs classified in the list of substances with narrow therapeutic margins.

## Conclusion

The determinants of generic substitution are numerous. Patients' health status (age and complexity of treatment) explain a part of the reluctance to substitute an original formulation by a generic, but this uncontrollable determinant is responsible for only a tiny part of substitution rate variations. The economic incentives (deductible and out-of-pocket participation of patients, number of generics sold on the Swiss market) were efficient, but with a moderate global effect. In contrast, adopting the practices of the canton with the highest generic use would itself increase the substitution rate from up to 26%. There was also evidence that an abundant supply of generics would also provide strong incentives to shift towards generics.

## List of abbreviations used

AIC: Akaike's information criterion; ATC: Anatomical Therapeutic Chemical; BIC: Schwarz's Bayesian information criterion; CI: Confidence interval; NTI: Narrow therapeutic index; OECD: Organisation for Economic Co-operation and Development; OTC: Over-the-counter; USA: United States of America

## Competing interests

The authors declare that they have no competing interests.

## Authors' contributions

All the four authors participated to the design of the study, to the statistical analysis, interpretation of results, to the writing of the paper and approved the final manuscript.

## Pre-publication history

The pre-publication history for this paper can be accessed here:

http://www.biomedcentral.com/1472-6963/11/17/prepub
